# Induction and characterization of pancreatic cancer in a transgenic pig model

**DOI:** 10.1371/journal.pone.0239391

**Published:** 2020-09-21

**Authors:** F. Edward Boas, Fuad Nurili, Achiude Bendet, Christopher Cheleuitte-Nieves, Olca Basturk, Gokce Askan, Adam O. Michel, Sebastien Monette, Etay Ziv, Constantinos T. Sofocleous, Aaron W. P. Maxwell, Lawrence B. Schook, Stephen B. Solomon, David P. Kelsen, Avigdor Scherz, Hooman Yarmohammadi

**Affiliations:** 1 Interventional Radiology Service, Department of Radiology, Memorial Sloan Kettering Cancer Center, New York, New York, United States of America; 2 Research Animal Resource Center, Sloan Kettering Institute, New York, New York, United States of America; 3 Department of Pathology, Memorial Sloan Kettering Cancer Center, New York, New York, United States of America; 4 Laboratory of Comparative Pathology, Memorial Sloan Kettering Cancer Center, The Rockefeller University, Weill Cornell Medicine, New York, New York, United States of America; 5 Department of Animal Sciences, University of Illinois at Urbana-Champaign, Urbana, Illinois, United States of America; 6 Department of Medicine, Memorial Sloan Kettering Cancer Center, New York, New York, United States of America; 7 Department of Plant and Environmental Sciences, Weizmann Institute of Science, Rehovot, Israel; Centro Nacional de Investigaciones Oncologicas, SPAIN

## Abstract

**Background:**

Preclinical testing of new locoregional therapies for pancreatic cancer has been challenging, due to the lack of a suitable large animal model.

**Purpose:**

To develop and characterize a porcine model of pancreatic cancer. Unlike small animals, pigs have similar physiology, drug dosing, and immune response to humans. Locoregional therapy in pigs can be performed using the same size catheters and devices as in humans.

**Methods:**

The Oncopig is a transgenic pig with Cre-inducible *TP53*^*R167H*^ and *KRAS*^*G12D*^ mutations. In 12 Oncopigs, CT-guided core biopsy of the pancreas was performed. The core biopsy was incubated with an adenoviral vector carrying the Cre recombinase gene. The transformed core biopsy was injected back into the pancreas (head, tail, or both). The resulting tumors (*n* = 19) were characterized on multi-phase contrast-enhanced CT, and on pathology, including immunohistochemistry. Angiographic characterization of the tumors was performed in 3 pigs.

**Results:**

Pancreatic tumors developed at 19 out of 22 sites (86%) that were inoculated. Average tumor size was 3.0 cm at 1 week (range: 0.5–5.1 cm). H&E and immunohistochemical stains revealed undifferentiated carcinomas, similar to those of the pancreatobiliary system in humans. Neoplastic cells were accompanied by a major inflammatory component. 1 of 12 pigs only had inflammatory nodules without evidence of neoplasia. On multiphase CT, tumors were hypovascular compared to the normal pancreas. There was no pancreatic duct dilation. In 3 pigs, angiography was performed, and in all 3 cases, the artery supplying the pancreatic tumor could be catheterized using a 2.4 F microcatheter. Selective angiography showed the pancreatic tumor, without extra-pancreatic perfusion.

**Conclusion:**

Pancreatic cancer can be induced in a transgenic pig. Intra-arterial procedures using catheters designed for human interventions were technically feasible in this large animal model.

## Introduction

Pancreatic cancer deaths are increasing, and are projected to become the second most common cause of cancer-related death in the United States by 2030 [1]. The 5-year survival rate is 6% [2]. Fewer than 20% of patients are resectable, and 80% of patients have recurrent disease after resection [3]. Better therapies are desperately needed.

Current locoregional therapies for pancreatic cancer are suboptimal. Irreversible electroporation of locally advanced pancreatic cancer can be performed, with a major complication rate of 40%, and median overall survival of 11 months [4]. Liver metastases from pancreatic cancer can be treated using ablation, embolization, or radioembolization, and although many patients respond radiographically, progression is rapid, and overall survival is less than 9 months [5].

Several new locoregional therapies for pancreatic cancer have been proposed, including local drug delivery using ultrasound microbubbles [6, 7], pancreatic transarterial chemoinfusion [8–11], pancreatic chemoembolization using lipiodol [12] or drug-eluting beads [13], intratumoral injection of oncolytic virus [14], and peritumoral injection of siRNA [15]. Translation to human trials has been challenging.

Due to the lack of a suitable large animal model of pancreatic cancer, many new locoregional therapies for pancreatic cancer are initially tested in normal pig pancreas [9, 11], or in nude mice with subcutaneous [6] or orthotopic [15] pancreatic tumor xenografts. Normal pancreas is not an ideal model for pancreatic cancer. Nude mice are immunocompromised, and require much smaller devices and catheters than humans. Furthermore, new cancer therapies developed in rodents have a high failure rate when translated to humans [16]. Presumably this is due to differences in physiology [17, 18], drug dosing [19, 20], and immune response [21–24] between rodents and humans.

Here, we develop and characterize a new immunocompetent pig model of pancreatic cancer. This model allows us to test new locoregional therapies for pancreatic cancer that are not yet ready for human trials.

## Methods

### Animals

All research procedures were approved by the Institutional Animal Care and Use Committee at Memorial Sloan Kettering Cancer Center. Our animal facility is AAALAC accredited and operates in compliance with the Guide for the Care and Use of Laboratory Animals [25]. Euthanasia was performed by administering pentobarbital sodium and phenytoin sodium solution (Euthasol, Virbac, Forth Worth, TX) intravenously.

12 female Oncopigs were obtained from the University of Illinois, or the National Swine Resource and Research Center at the University of Missouri. Oncopigs are transgenic pigs with Cre-recombinase-inducible heterozygous *TP53*^*R167H*^ and *KRAS*^*G12D*^ mutations [26, 27]. R167H is a dominant-negative mutation of the *TP53* tumor suppressor gene, and G12D is an activating mutation of the *KRAS* oncogene.

Animals were maintained in pens with aspen-chip contact bedding (PWI Industries Canada, Quebec, Canada), fed a grower chow (#5081, PMI, St Louis, MO), and provided water ad libitum. Animal room temperature was 21.5±1°C, relative humidity was 30%– 70%, and light:dark photoperiod was 12:12 hours. All procedures and imaging were performed under general anesthesia, with peri-operative analgesia.

### Tumor induction

Tumor induction was performed when the pigs were 12–22 weeks old. An 18 gauge core biopsy of the pancreas was obtained under CT guidance, using co-axial technique (Temno Evolution, Merit Medical, South Jordan, UT). *TP53*^*R167H*^ and *KRAS*^*G12D*^ expression was induced by incubating the core biopsy with an adenoviral vector carrying the Cre recombinase gene (10^9^ pfu Ad5CMVCre-eGFP, University of Iowa Viral Vector Core) for 20 minutes at room temperature, in phosphate-buffered saline containing 15 mM calcium chloride (total fluid volume of 1 ml). Gelatin sponge (Gelfoam, Pfizer) was then added using a 3-way stopcock, and the mixture (virus, core biopsy, gelatin) was injected percutaneously back into the duodenal or splenic lobe of the pancreas, through the biopsy needle, which was kept in place after the biopsy. Note that pigs have a ring-shaped pancreas with 3 lobes: duodenal, splenic, and connecting. In this paper, we will refer to the duodenal lobe as the “head” of the pancreas, and the splenic lobe as the “tail.”

### Multiphase contrast enhanced CT

Five-phase contrast-enhanced CT was performed 1 week after tumor inoculation. Non-contrast CT of the abdomen and pelvis was obtained. Omnipaque 300 (2 ml/kg, max 150 ml) was power injected at 2–3 ml/sec. The early arterial phase CT scan was obtained when the abdominal aorta reached 150 Hounsfield units. The late arterial phase was obtained 15 seconds after the early arterial phase. The portal venous phase was obtained 25 seconds after the late arterial phase scan. The delayed phase scan was obtained 90 seconds after portal venous phase. All scans were obtained at 120 kVp. Mean Hounsfield units of the tumor, normal pancreas, and aorta were measured on each phase, using elliptical regions of interest (ROI).

### Angiography and cone beam CT

Angiography was performed 1 week after tumor inoculation. From femoral access, under fluoroscopic guidance, a 4 F catheter was advanced into the celiac artery, and an arteriogram was performed. A 2.4 F microcatheter (Merit Maestro, Infiniti Medical, Redwood City, CA) was advanced into a pancreatic artery, and an arteriogram was performed. Cone beam CT arteriogram was obtained during a breath hold after administering a paralytic agent (rocuronium 1–1.6 mg/kg IV).

### Pathology

Two weeks after inoculation, animals were euthanized, and tumors were macroscopically examined, harvested, and fixed in 10% neutral buffered formalin. Following formalin fixation, sections of tumor were processed into paraffin blocks, and sectioned at 5 micron thickness. Hematoxylin and eosin (H&E)-stained sections were reviewed by both human (OB, GA) and veterinary (AOM, SM) pathologists. Representative formalin-fixed paraffin-embedded tissue sections were immunolabeled with antibodies against cytokeratin AE1/AE3, cytokeratin 8/18, vimentin, Iba1, and CD31, as described in S1 Table. Masson’s trichome stain for collagen was also performed.

## Results

Pancreatic tumors developed at 19 out of 22 sites (86%) that were inoculated. Average tumor size was 3.0 cm at 1 week (range: 0.5–5.1 cm). There were no complications from the tumor inoculation procedure, based on daily clinical evaluation and imaging.

**Fig 1:**
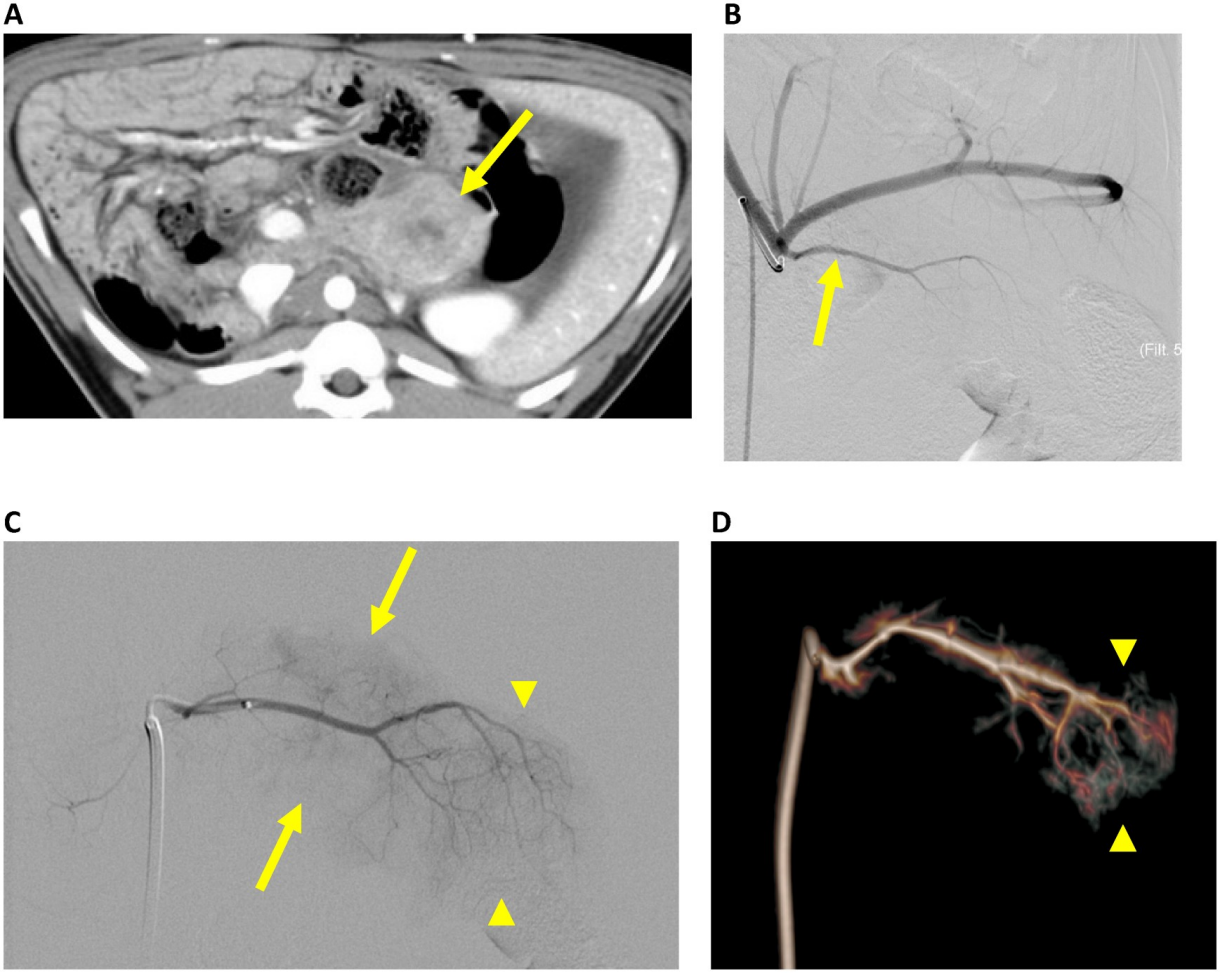
CT and catheter angiography of a pig pancreatic tumor. **A**. Contrast-enhanced CT shows a tumor in the tail of the pancreas (arrow). **B**. Celiac arteriogram shows the dorsal pancreatic artery (arrow). **C**. Dorsal pancreatic arteriogram shows an enhancing pancreatic mass (arrows) supplied by tiny branches (430 μm or smaller) of the proximal dorsal pancreatic artery, while larger distal branches supply the normal tail of the pancreas (arrowheads). **D**. Cone beam CT arteriogram shows the dorsal pancreatic artery supplying the tumor and the tail of the pancreas (arrowheads), without extrapancreatic perfusion. The pancreatic tumor appears hypovascular, compared to the normal pancreas.

**Fig 2:**
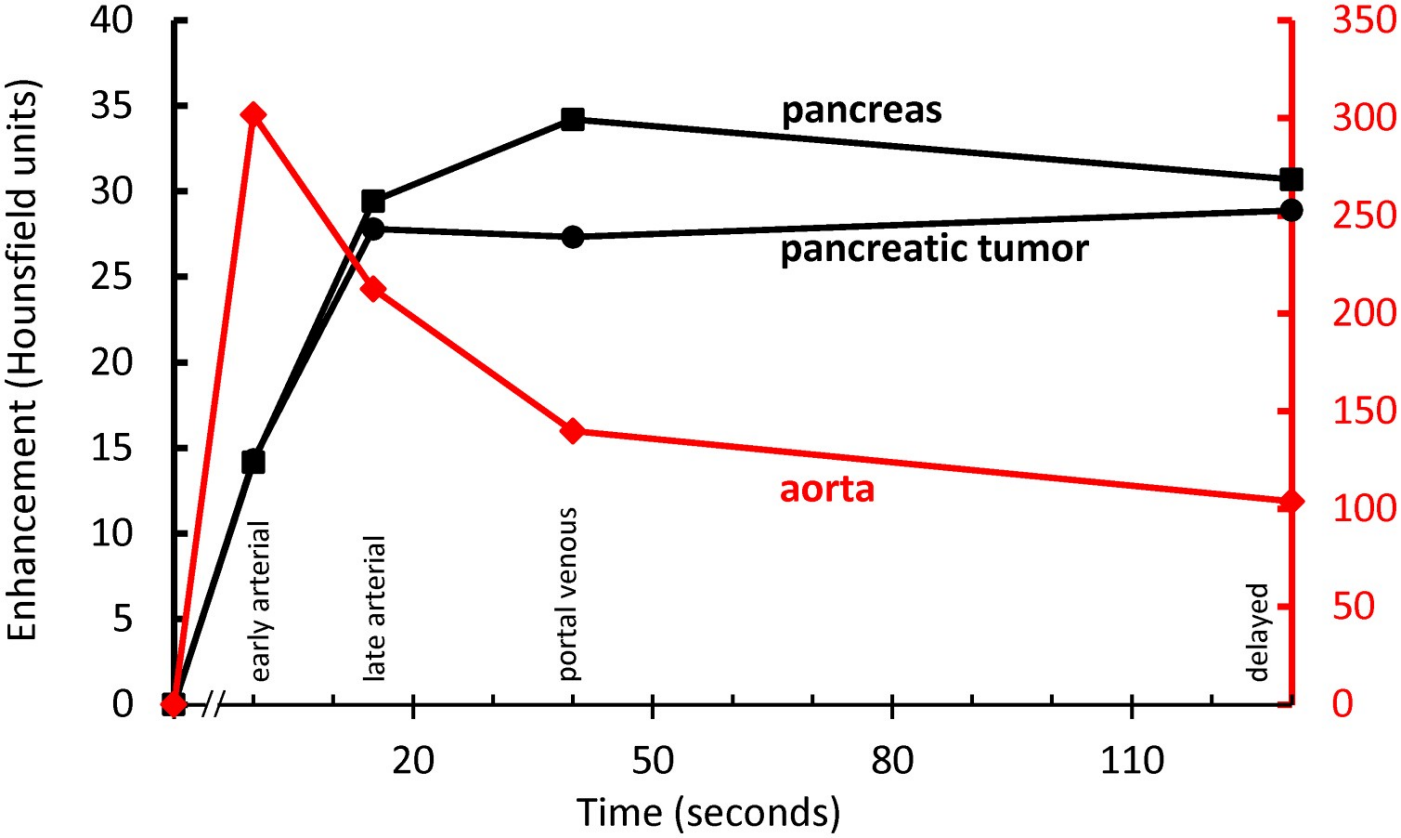
Average enhancement curves of normal pancreas (primary *y*-axis), pancreatic tumors (primary *y*-axis), and aorta (secondary *y*-axis). The pancreatic tumors are hypoenhancing, compared to normal pancreas, in the portal venous phase.

On multiphase CT, tumors were enhancing (Fig 1A and 1C), but hypovascular compared to the normal pancreas (Fig 2). There was no pancreatic or biliary duct dilation.

In 3 pigs, angiography was performed, and in all 3 cases, the dorsal pancreatic artery supplied the pancreatic tail tumors. In all cases, the dorsal pancreatic artery could be selected using a 2.4 F microcatheter, and selective angiography showed the pancreatic tumor, without extra-pancreatic perfusion (Fig 1B–1D).

**Fig 3:**
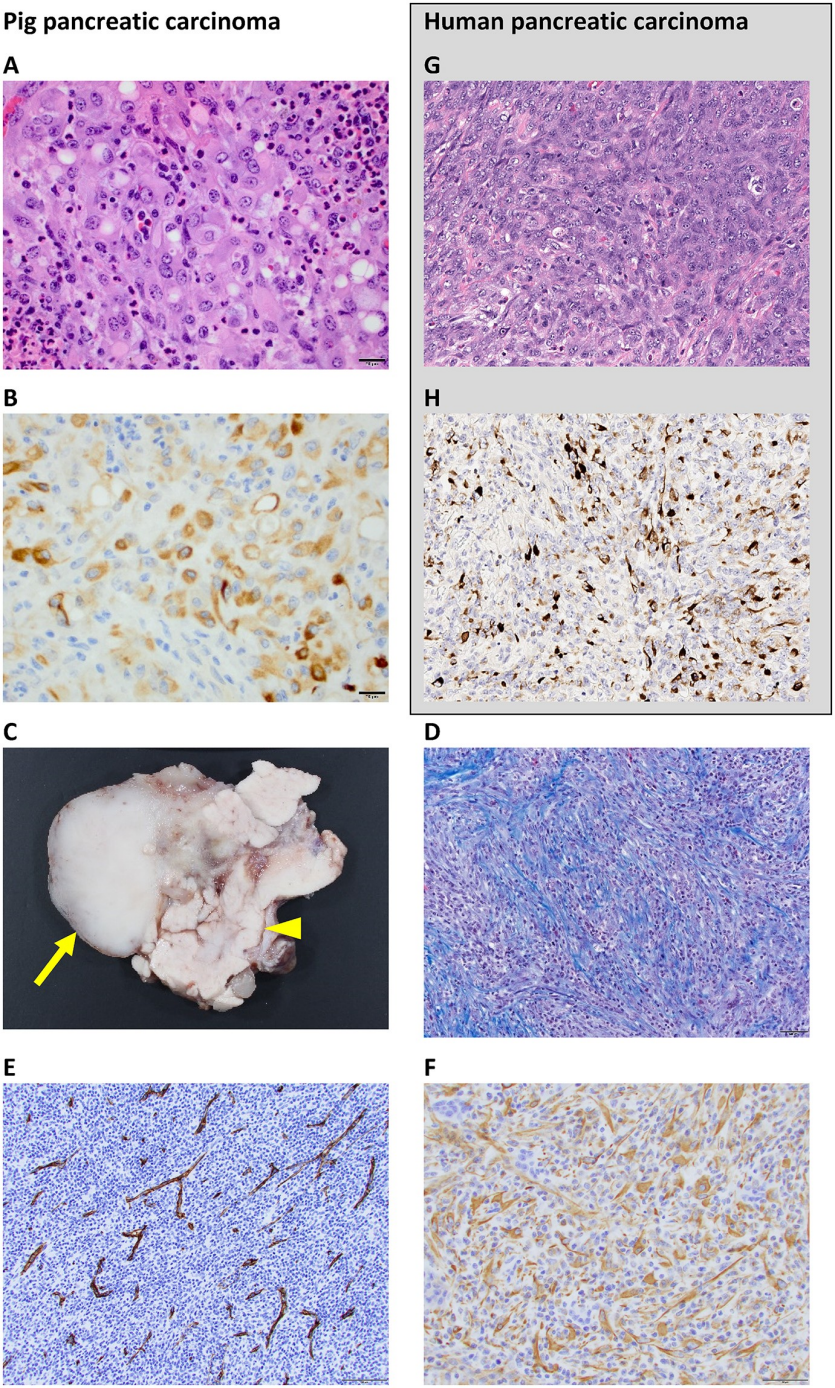
Pathology of pig pancreatic tumors. (**A**) H&E stained section reveals sheets of atypical epithelioid cells with eosinophilic cytoplasm and large round to oval nuclei as well as an associated inflammatory component. (**B**) Cytokeratin AE1/AE3 expression confirms epithelioid differentiation. (**C**) Gross pathology shows a solid mass (arrow) arising from the pancreas (arrowhead). (**D**) Masson’s trichrome stain shows collagen bundles (blue) within tumor stroma. (**E**) CD31 immunohistochemistry shows that the tumors contain a high density of microscopic blood vessels. (**F**) Vimentin immunohistochemistry shows that spindle cells are immunopositive, indicating mesenchymal differentiation. (**G** and **H**) For comparison, H&E stain and cytokeratin-19 immunohistochemistry of an undifferentiated carcinoma of the human pancreas shows similar morphologic features.

Grossly, the tumor were soft, poorly demarcated, pale-tan lesions located within the pancreatic parenchyma. In 11 of 12 pigs, H&E (Fig 3) showed undifferentiated carcinomas composed of sheets of epithelioid cells and streams of spindle cells, with or without multinucleated giant cells, resembling undifferentiated carcinomas of the pancreatobiliary tract in humans [28, 29]. Neoplastic cells were accompanied by a major inflammatory component in all tumors. One of 12 pigs only had inflammatory nodules, without evidence of a neoplastic process.

On immunohistochemistry (Fig 3), epithelioid cells were strongly immunopositive for cytokeratin AE1/AE3 and minimally immunopositive for 8/18, indicating epithelial differentiation, and spindle cells were immunopositive for vimentin, indicating mesenchymal differentiation. The spindle cells in these carcinomas are likely due to epithelial-to-mesenchymal transition. Giant cells were negative for cytokeratins and immunopositive for Iba1, confirming histiocytic origin. In the carcinomas, Masson’s trichrome stain highlighted a collagen-containing desmoplastic stroma, but the amount of the stroma was significantly less compared to that of human pancreatic ductal adenocarcinoma. Tumor angiogenesis was shown on CD31 stains. The tumors were supplied by tiny vessels, which were seen both on angiography (Fig 1) and immunohistochemistry (Fig 3E).

## Discussion

We have developed and characterized a pig model of pancreatic cancer. Tumor inoculation is simple, reproducible, and site-specific, and results in rapidly growing undifferentiated carcinomas with a major inflammatory component, similar to the pancreatobiliary carcinomas seen in humans. Oncopig pancreatic cancer contains both *TP53* and *KRAS* mutations, which are among the most common mutations seen in human pancreatic ductal adenocarcinoma.

Unlike mice and other small animals, pigs have similar physiology [17, 18], drug dosing [19, 20], and immune response [21–24] to humans. Locoregional therapy in pigs can be performed using the same size catheters and devices as humans. The artery supplying pig pancreatic cancer could be selectively catheterized using a standard 2.4 F microcatheter. Thus, the Oncopig pancreatic tumor model can be used to develop new image-guided therapies, such as transarterial embolization [30], local immunotherapy [31], and vascular targeted photodynamic therapy [32].

Oncopig pancreatic tumors recreate some of the key challenges for drug delivery in pancreatic cancer: like pancreatic adenocarcinoma in humans, Oncopig pancreatic tumors are hypovascular, and are supplied by tiny feeding arteries [8]. Thus, Oncopig pancreatic cancer could be a promising new model system to test therapies that overcome these barriers to local drug delivery.

One important component of the tumor inoculation protocol is the use of gelatin sponge to retain virus and tumor cells at the site of injection, and to create a receptive microenvironment for tumor growth. Previously, we showed that direct injection of adenoviral vector into the Oncopig, without gelatin sponge, did not result in tumor development [33]. Tumor cells alone are often insufficient for tumor growth, without a receptive microenvironment [34]. For example, in the rabbit VX2 model, tumors must first be grown subcutaneously, prior to transplanting the tumor into the liver or pancreas [35]. Here, we show that direct inoculation of a solid organ is possible, using gelatin sponge, which is made from collagen, an important part of the extracellular matrix in tumors.

A major inflammatory component was seen in all of the Oncopig pancreatic tumors. Undifferentiated carcinomas in humans can also contain significant inflammation [28, 29]. Subcutaneous and intramuscular tumors in the Oncopig contain a significant inflammatory component, which is due to an antitumor T-cell response [36]. Future experiments should address whether these inflammatory pig tumors serve as a good model for the anti-tumor immune response in humans.

Several other animal models of pancreatic cancer are available [37]. The KPC mouse model of pancreatic adenocarcinoma [38] can be used to test drugs, but mice are too small to use human ablation probes or catheters. VX2 tumors can be implanted in rabbit pancreas, and the GDA can be catheterized, but selective angiography of a pancreatic artery has not been reported in rabbits [35]. A previously reported Oncopig pancreatic cancer model used a surgical (not percutaneous) inoculation technique, and required 1 year for growth of small pancreatic tumors that were not visible on computed tomography [39]. The prior Oncopig paper also reported development of large tumors (described as leiomyosarcomas) 16 days after inoculation, but these tumors were not characterized radiographically. In this paper, we solve some technical challenges with solid organ tumor induction in the Oncopig, and report the first large animal pancreatic cancer model that enables testing of new intra-arterial therapies.

One limitation of the Oncopig model is that there is no pancreatic duct dilation. Another limitation is that the inflammatory, poorly differentiated, rapidly growing tumors might not be a good model for well differentiated or slowly growing tumors.

In conclusion, Oncopig pancreatic tumors are rapidly growing, immunogenic, hypovascular undifferentiated carcinomas that can be used to test new percutaneous and intra-arterial therapies for pancreatic cancer.

## Supporting information

S1 Table (DOCX)

S2 Table (DOCX)

S3 Table (DOCX)

## References

[1] Rahib L, Smith BD, Aizenberg R, Rosenzweig AB, Fleshman JM, Matrisian LM. Projecting cancer incidence and deaths to 2030: the unexpected burden of thyroid, liver, and pancreas cancers in the United States. Cancer Res. 2014;74(11):2913–21. Epub 2014/05/21. 10.1158/0008-5472.CAN-14-0155 . 24840647

[2] Ilic M, Ilic I. Epidemiology of pancreatic cancer. World J Gastroenterol. 2016;22(44):9694–705. Epub 2016/12/14. 10.3748/wjg.v22.i44.9694 . 27956793 PMC5124974

[3] Kleeff J, Korc M, Apte M, La Vecchia C, Johnson CD, Biankin AV, et al Pancreatic cancer. Nat Rev Dis Primers. 2016;2:16022 Epub 2016/05/10. 10.1038/nrdp.2016.22 . 27158978

[4] Scheffer HJ, Vroomen LG, de Jong MC, Melenhorst MC, Zonderhuis BM, Daams F, et al Ablation of Locally Advanced Pancreatic Cancer with Percutaneous Irreversible Electroporation: Results of the Phase I/II PANFIRE Study. Radiology. 2017;282(2):585–97. Epub 2016/09/08. 10.1148/radiol.2016152835 . 27604035

[5] Yoon H, Mandel JE, Zener R, Yarmohammadi H, Solomon SB, Sofocleous CT, et al., editors. Outcomes after locoregional therapy of pancreatic adenocarcinoma liver metastases. WCIO; 2018; Boston, MA.

[6] Gao F, Wu J, Niu S, Sun T, Li F, Bai Y, et al Biodegradable, pH-Sensitive Hollow Mesoporous Organosilica Nanoparticle (HMON) with Controlled Release of Pirfenidone and Ultrasound-Target-Microbubble-Destruction (UTMD) for Pancreatic Cancer Treatment. Theranostics. 2019;9(20):6002–18. Epub 2019/09/20. 10.7150/thno.36135 . 31534533 PMC6735371

[7] Dimcevski G, Kotopoulis S, Bjanes T, Hoem D, Schjott J, Gjertsen BT, et al A human clinical trial using ultrasound and microbubbles to enhance gemcitabine treatment of inoperable pancreatic cancer. J Control Release. 2016;243:172–81. Epub 2016/11/05. 10.1016/j.jconrel.2016.10.007 . 27744037

[8] Rosemurgy AS, Ross SB, Vitulli PL, Malek R, Li J, Agah R. Safety Study of Targeted and Localized Intra-Arterial Delivery of Gemcitabine in Patients with Locally Advanced Pancreatic Adenocarcinoma. J Pancreat Cancer. 2017;3(1):58–65. Epub 2017/08/01. 10.1089/pancan.2017.0011 . 30631844 PMC5933492

[9] Murata S, Onozawa S, Mine T, Ueda T, Sugihara F, Yasui D, et al Minimizing Systemic Leakage of Cisplatin during Percutaneous Isolated Pancreas Perfusion Chemotherapy: A Pilot Study. Radiology. 2015;276(1):102–9. Epub 2015/03/04. 10.1148/radiol.15141596 . 25734552

[10] Tanaka T, Sakaguchi H, Sho M, Yamamoto K, Nishiofuku H, Nakajima Y, et al A novel interventional radiology technique for arterial infusion chemotherapy against advanced pancreatic cancer. AJR Am J Roentgenol. 2009;192(4):W168–77. Epub 2009/03/24. 10.2214/AJR.08.1392 . 19304677

[11] Murata S, Onozawa S, Yasui D, Ueda T, Sugihara F, Shimizu A, et al Evaluating the Feasibility of Isolated Pancreatic Perfusion for Chemotherapy Using Computed Tomography: An Experimental Study in Pig Models. Cardiovasc Intervent Radiol. 2018;41(7):1081–8. Epub 2018/03/28. 10.1007/s00270-018-1943-y . 29582129

[12] Yoshida H, Onda M, Tajiri T, Uchida E, Arima Y, Mamada Y, et al Experience with intraarterial infusion of styrene maleic acid neocarzinostatin (SMANCS)-lipiodol in pancreatic cancer. Hepatogastroenterology. 1999;46(28):2612–5. Epub 1999/10/16. . 10522050

[13] Chick JF, Chen JX, Bennett SJ, Chauhan NR, Reddy SN, Gade T, et al Incidental Regression of a Suspected Pancreatic Intraductal Papillary Mucinous Neoplasm after Nontarget Embolization. J Vasc Interv Radiol. 2016;27(6):922–3. Epub 2016/06/12. 10.1016/j.jvir.2015.12.758 . 27287972

[14] Hirooka Y, Kasuya H, Ishikawa T, Kawashima H, Ohno E, Villalobos IB, et al A Phase I clinical trial of EUS-guided intratumoral injection of the oncolytic virus, HF10 for unresectable locally advanced pancreatic cancer. BMC Cancer. 2018;18(1):596 Epub 2018/05/29. 10.1186/s12885-018-4453-z . 29801474 PMC5970460

[15] Lei Y, Tang L, Xie Y, Xianyu Y, Zhang L, Wang P, et al Gold nanoclusters-assisted delivery of NGF siRNA for effective treatment of pancreatic cancer. Nat Commun. 2017;8:15130 Epub 2017/04/26. 10.1038/ncomms15130 . 28440296 PMC5414062

[16] Mak IW, Evaniew N, Ghert M. Lost in translation: animal models and clinical trials in cancer treatment. Am J Transl Res. 2014;6(2):114–8. Epub 2014/02/04. . 24489990 PMC3902221

[17] West GB, Brown JH, Enquist BJ. A general model for the origin of allometric scaling laws in biology. Science. 1997;276(5309):122–6. Epub 1997/04/04. 10.1126/science.276.5309.122 . 9082983

[18] Schmidt-Nielsen K. Scaling: Why is animal size so important? New York: Cambridge University Press; 1984.

[19] Nair AB, Jacob S. A simple practice guide for dose conversion between animals and human. J Basic Clin Pharm. 2016;7(2):27–31. Epub 2016/04/09. 10.4103/0976-0105.177703 . 27057123 PMC4804402

[20] Sharma V, McNeill JH. To scale or not to scale: the principles of dose extrapolation. Br J Pharmacol. 2009;157(6):907–21. Epub 2009/06/11. 10.1111/j.1476-5381.2009.00267.x . 19508398 PMC2737649

[21] Overgaard NH, Frosig TM, Welner S, Rasmussen M, Ilsoe M, Sorensen MR, et al Establishing the pig as a large animal model for vaccine development against human cancer. Front Genet. 2015;6:286 Epub 2015/10/07. 10.3389/fgene.2015.00286 . 26442104 PMC4584933

[22] Dawson HD, Smith AD, Chen C, Urban JF Jr. An in-depth comparison of the porcine, murine and human inflammasomes; lessons from the porcine genome and transcriptome. Vet Microbiol. 2017;202:2–15. Epub 2016/06/21. 10.1016/j.vetmic.2016.05.013 . 27321134

[23] Dawson HD, Loveland JE, Pascal G, Gilbert JG, Uenishi H, Mann KM, et al Structural and functional annotation of the porcine immunome. BMC Genomics. 2013;14:332 Epub 2013/05/17. 10.1186/1471-2164-14-332 . 23676093 PMC3658956

[24] Seok J, Warren HS, Cuenca AG, Mindrinos MN, Baker HV, Xu W, et al Genomic responses in mouse models poorly mimic human inflammatory diseases. Proc Natl Acad Sci U S A. 2013;110(9):3507–12. Epub 2013/02/13. 10.1073/pnas.1222878110 . 23401516 PMC3587220

[25] National Research Council (U.S.) Committee for the Update of the Guide for the Care and Use of Laboratory Animals, Institute for Laboratory Animal Research (U.S.), (U.S.) NAP. Guide for the care and use of laboratory animals. Washington, D.C.: National Academies Press; 2011. http://www.ncbi.nlm.nih.gov/books/NBK54050.

[26] Schook LB, Collares TB, Hu W, Liang Y, Rodrigues FM, Rund LA, et al A genetic porcine model of cancer. PLOS One. 2015;10(7):e0128864 10.1371/journal.pone.0128864 26132737 PMC4488487

[27] Nurili F, Monette S, Michel AO, Bendet A, Basturk O, Askan G, et al Transarterial embolization of liver cancer in a transgenic pig model. Journal of vascular and interventional radiology. 2020;Submitted. 10.1016/j.jvir.2020.09.011 PMC8451249 33500185

[28] Muraki T, Reid MD, Basturk O, Jang KT, Bedolla G, Bagci P, et al Undifferentiated Carcinoma With Osteoclastic Giant Cells of the Pancreas: Clinicopathologic Analysis of 38 Cases Highlights a More Protracted Clinical Course Than Currently Appreciated. Am J Surg Pathol. 2016;40(9):1203–16. Epub 2016/08/11. 10.1097/PAS.0000000000000689 . 27508975 PMC4987218

[29] Reid MD, Basturk O, Thirabanjasak D, Hruban RH, Klimstra DS, Bagci P, et al Tumor-infiltrating neutrophils in pancreatic neoplasia. Mod Pathol. 2011;24(12):1612–9. Epub 2011/08/09. 10.1038/modpathol.2011.113 . 21822201 PMC3227778

[30] Yarmohammadi H, Gonzalez-Aguirre AJ, Schook L, Ziv E, Erinjeri JP, Brown KT, et al Treatment of pancreatic cancer by intra-arterial injection of an emulsion of Lipiodol and Bumetanide (an anti-glycolytic drug) in a porcine model: Initial results. JVIR. 2017;28(2):S8–9.

[31] Boas FE, Nurili F, Erinjeri JP, Schook LB, Solomon SB, Yarmohammadi H. Local immunotherapy: Intra-arterial liver tumor vaccination in a pig model of metastatic pancreatic cancer. JVIR. 2019;30(3S):S98–9.

[32] Yarmohammadi H, Nurili F, Fujimori M, Monette S, Kelsen D, Coleman J, et al Nonthermal ablation of pancreatic cancer in a pig model, using vascular-targeted photodynamic therapy (VTP). JVIR. 2019;30(3S):S266.

[33] Boas F, Gonzalez Aguirre A, Srimathveeravalli G, Rund L, Schwind R, Schook L, et al Induction of pancreatic cancer in a porcine model: Initial results. J Vasc Interv Radiol. 2017;28(2):S181.

[34] Wang M, Zhao J, Zhang L, Wei F, Lian Y, Wu Y, et al Role of tumor microenvironment in tumorigenesis. J Cancer. 2017;8(5):761–73. Epub 2017/04/07. 10.7150/jca.17648 . 28382138 PMC5381164

[35] Eifler AC, Lewandowski RJ, Virmani S, Chung JC, Wang D, Tang RL, et al Development of a VX2 pancreatic cancer model in rabbits: a pilot study. J Vasc Interv Radiol. 2009;20(8):1075–82. Epub 2009/06/30. 10.1016/j.jvir.2009.04.051 . 19560941 PMC2917605

[36] Overgaard NH, Principe DR, Schachtschneider KM, Jakobsen JT, Rund LA, Grippo PJ, et al Genetically Induced Tumors in the Oncopig Model Invoke an Antitumor Immune Response Dominated by Cytotoxic CD8beta(+) T Cells and Differentiated gammadelta T Cells Alongside a Regulatory Response Mediated by FOXP3(+) T Cells and Immunoregulatory Molecules. Front Immunol. 2018;9:1301 Epub 2018/06/23. 10.3389/fimmu.2018.01301 . 29930558 PMC5999797

[37] Kalla D, Kind A, Schnieke A. Genetically Engineered Pigs to Study Cancer. Int J Mol Sci. 2020;21(2). Epub 2020/01/17. 10.3390/ijms21020488 . 31940967 PMC7013672

[38] Lee JW, Komar CA, Bengsch F, Graham K, Beatty GL. Genetically Engineered Mouse Models of Pancreatic Cancer: The KPC Model (LSL-Kras(G12D/+); LSL-Trp53(R172H/+); Pdx-1-Cre), Its Variants, and Their Application in Immuno-oncology Drug Discovery. Curr Protoc Pharmacol. 2016;73:14 39 1–14 39 20. Epub 2016/06/02. 10.1002/cpph.2 . 27248578 PMC4915217

[39] Principe DR, Overgaard NH, Park AJ, Diaz AM, Torres C, McKinney R, et al KRAS(G12D) and TP53(R167H) Cooperate to Induce Pancreatic Ductal Adenocarcinoma in Sus scrofa Pigs. Sci Rep. 2018;8(1):12548 Epub 2018/08/24. 10.1038/s41598-018-30916-6 . 30135483 PMC6105629

