# Supplementary material for: Induction and characterization of pancreatic cancer in a transgenic pig model
Source: PLoS One. 2020 Sep 21;15(9):e0239391. doi: 10.1371/journal.pone.0239391 (PMC7505440; doi:10.1371/journal.pone.0239391)
Supplement: S1 Table — (DOCX) [file pone.0239391.s001.docx]

Boas FE, et al. (2020) “Induction and characterization of pancreatic cancer in a transgenic pig model.”

| **Marker** | **Staining platform** | **Epitope retrieval** | **Primary antibody** | **Primary antibody concentration** | **Secondary antibody and detection system** |
| --- | --- | --- | --- | --- | --- |
| Cytokeratin AE1/AE3 | Ventana XT,  Roche | Tris EDTA based  CC1 retrieval  (Ventana), pH 8.0 | Ventana-  Roche  760-2135 | Prediluted,  1:5 | Vector Labs mkb-  2225, Ventana DAB  MAP (Roche) |
| Cytokeratin 8/18 | Manual | Heat induced, pH 6.0 | Fitzgerald 2-R-CP004 | 1:1500 | Vector Laboratories BA-7000, PK-6100 |
| Vimentin | Leica Bond RX | Heat induced, pH 9.0 | Cell Signaling 5741 | 1:250 | Leica Biosystems, DS9800 |
| Iba1 | Leica Bond RX | Heat induced, pH 9.0 | Abcam ab5076 | 1:500 | Vector Laboratories BA-5000, Leica Biosystems, DS9800 |
| CD31 | Manual | Heat induced, pH 9.0 | Abcam ab28364 | 1:100 | Vector Laboratory BA-1000, PK-6100 |

**Supplemental Table 1**. Immunohistochemistry technique.
