# Supplementary material for: Induction and characterization of pancreatic cancer in a transgenic pig model
Source: PLoS One. 2020 Sep 21;15(9):e0239391. doi: 10.1371/journal.pone.0239391 (PMC7505440; doi:10.1371/journal.pone.0239391)
Supplement: S2 Table — (DOCX) [file pone.0239391.s002.docx]

| **Site number** | **Litter number** | **Pig number** | **Age at inoculation (days)** | **Sex** | **Site** | **Tumor diameter on CT at 1 week (cm)** | **Histology** |
| --- | --- | --- | --- | --- | --- | --- | --- |
| 1 | 1 | 1 | 157 | F | pancreas (head) | no tumor |  |
| 2 | 1 | 1 | 157 | F | pancreas (tail) | 3.1 | undifferentiated carcinoma |
| 3 | 2 | 2 | 116 | F | pancreas (tail) | no tumor |  |
| 4 | 2 | 3 | 137 | F | pancreas (head) | 3.2 | undifferentiated carcinoma |
| 5 | 2 | 4 | 137 | F | pancreas (head) | 5 | undifferentiated carcinoma |
| 6 | 2 | 4 | 137 | F | pancreas (tail) | 5.1 | undifferentiated carcinoma |
| 7 | 3 | 5 | 87 | F | pancreas (head) | 3.6 | undifferentiated carcinoma |
| 8 | 3 | 5 | 87 | F | pancreas (tail) | 3.5 | undifferentiated carcinoma |
| 9 | 3 | 6 | 101 | F | pancreas (head) | 2.9 | undifferentiated carcinoma |
| 10 | 3 | 6 | 101 | F | pancreas (tail) | 3.2 | undifferentiated carcinoma |
| 11 | 3 | 7 | 102 | F | pancreas (head) | 3.3 | undifferentiated carcinoma |
| 12 | 3 | 7 | 102 | F | pancreas (tail) | 3.1 | undifferentiated carcinoma |
| 13 | 3 | 8 | 102 | F | pancreas (head) | no tumor |  |
| 14 | 3 | 8 | 102 | F | pancreas (tail) | 3.1 | undifferentiated carcinoma |
| 15 | 4 | 9 | 104 | F | pancreas (head) | 2.7 | undifferentiated carcinoma |
| 16 | 4 | 9 | 104 | F | pancreas (tail) | 3.7 | undifferentiated carcinoma |
| 17 | 4 | 10 | 111 | F | pancreas (head) | 1.4 | inflammation only |
| 18 | 4 | 10 | 111 | F | pancreas (tail) | 4 | inflammation only |
| 19 | 4 | 11 | 138 | F | pancreas (head) | 0.9 | inflammation only |
| 20 | 4 | 11 | 138 | F | pancreas (tail) | 1.5 | undifferentiated carcinoma |
| 21 | 4 | 12 | 152 | F | pancreas (head) | 0.5 | not evaluated |
| 22 | 4 | 12 | 152 | F | pancreas (tail) | 2.8 | undifferentiated carcinoma |

**Supplemental Table 2**. Pancreatic tumors.
