# Supplementary material for: Induction and characterization of pancreatic cancer in a transgenic pig model
Source: PLoS One. 2020 Sep 21;15(9):e0239391. doi: 10.1371/journal.pone.0239391 (PMC7505440; doi:10.1371/journal.pone.0239391)
Supplement: S3 Table — (DOCX) [file pone.0239391.s003.docx]

| **Phase** | **Time (sec)** | **Aortic enhancement** | **Pancreatic tumor enhancement** | **Pancreatic enhancement** |
| --- | --- | --- | --- | --- |
| non-contrast |  | 0 | 0 | 0 |
| early arterial | 0 | 301.7 | 14.3 | 14.2 |
| late arterial | 15 | 212.6 | 27.8 | 29.4 |
| portal venous | 40 | 139.9 | 27.3 | 34.2 |
| delayed | 130 | 103.9 | 28.9 | 30.7 |

**Supplemental Table 3**. Enhancement curves (Hounsfield units) from Figure 2.
